# Unexpected variability of gametogenic pathways and interspecific chromosomal leakage in *Pelophylax esculentus* hybrids^†^

**DOI:** 10.1093/biolre/ioag171

**Published:** 2026-08-19

**Authors:** Martyna Frątczak, Veronika Labajová, Eleonora Pustovalova, Zuzana Majtánová, Krzysztof Kolenda, Petr Papežík, Marie Doležálková-Kaštánková, Magdalena Chmielewska, Lukáš Choleva, Peter Mikulíček, Dmitrij Dedukh

**Affiliations:** Department of Zoology, Poznan University of Life Sciences, Poznań, Poland; Department of Biology and Ecology, Faculty of Science, University of Ostrava, Ostrava, Czech Republic; Laboratory of Fish Genetics, Institute of Animal Physiology and Genetics, The Czech Academy of Sciences, Liběchov, Czech Republic; Laboratory of Non-Mendelian Evolution, Institute of Animal Physiology and Genetics, The Czech Academy of Sciences, Liběchov, Czech Republic; Laboratory of Fish Genetics, Institute of Animal Physiology and Genetics, The Czech Academy of Sciences, Liběchov, Czech Republic; Laboratory of Non-Mendelian Evolution, Institute of Animal Physiology and Genetics, The Czech Academy of Sciences, Liběchov, Czech Republic; Laboratory of Fish Genetics, Institute of Animal Physiology and Genetics, The Czech Academy of Sciences, Liběchov, Czech Republic; Laboratory of Non-Mendelian Evolution, Institute of Animal Physiology and Genetics, The Czech Academy of Sciences, Liběchov, Czech Republic; Amphibian Biology Group, Department of Evolutionary Biology and Conservation of Vertebrates, Faculty of Biological Sciences, University of Wrocław, Wrocław, Poland; Department of Zoology, Faculty of Natural Sciences, Comenius University in Bratislava, Bratislava, Slovakia; Laboratory of Fish Genetics, Institute of Animal Physiology and Genetics, The Czech Academy of Sciences, Liběchov, Czech Republic; Laboratory of Non-Mendelian Evolution, Institute of Animal Physiology and Genetics, The Czech Academy of Sciences, Liběchov, Czech Republic; Amphibian Biology Group, Department of Evolutionary Biology and Conservation of Vertebrates, Faculty of Biological Sciences, University of Wrocław, Wrocław, Poland; Department of Biology and Ecology, Faculty of Science, University of Ostrava, Ostrava, Czech Republic; Laboratory of Fish Genetics, Institute of Animal Physiology and Genetics, The Czech Academy of Sciences, Liběchov, Czech Republic; Department of Zoology, Faculty of Natural Sciences, Comenius University in Bratislava, Bratislava, Slovakia; Laboratory of Non-Mendelian Evolution, Institute of Animal Physiology and Genetics, The Czech Academy of Sciences, Liběchov, Czech Republic

**Keywords:** bivalents, fluorescent in situ hybridization, hybridogenesis, meiosis, programmed DNA elimination, spermatogenesis, sterility

## Abstract

Hybridization is common among animals and plants, but often leads to sterility due to improper chromosome pairing. Some hybrids overcome sterility through modified gametogenesis, enabling clonal or hemiclonal reproduction. In water frog hybrids from the *Pelophylax esculentus* complex, hybridogenesis typically ensures hemiclonal reproduction. These hybrids inherit haploid chromosomal sets (n = 13) from both parental species, *P. lessonae* and *P. ridibundus*. During hybrid gametogenesis, one genome is eliminated while the other is clonally transmitted. We analyzed hybrid males and females from Central Europe, where hybrids coexist with *P. lessonae* and with both parental species, to assess gametogenic stability and whether the *P. lessonae* genome is consistently eliminated. Most hybrids followed canonical gametogenesis with premeiotic elimination of the *P. lessonae* genome and endoreplication of the *P. ridibundus* genome, yielding haploid *P. ridibundus* gametes. Nevertheless, gametogenesis was frequently altered in males but not in females. Some hybrid males had spermatocytes with 13 or 26 univalents, 26 bivalents, or tetravalents, indicating variability in genome elimination and endoreplication. Some males produced diploid or haploid spermatids with *P. ridibundus* and *P. lessonae* genomes. Comparative genome hybridization revealed that some males with altered gametogenesis carried unusual chromosomes with mixed R and L signals. Furthermore, rare triploid males showed substitutions of *P. lessonae* chromosomes to *P. ridibundus* chromosomes. Despite altered gametogenesis, they produced spermatocytes and haploid spermatids with *P. lessonae* chromosomes and two *P. ridibundus* chromosomes that escaped elimination. Overall, despite a generally consistent pattern of genome transmission, hybrid males exhibit frequent deviations from canonical hybridogenesis across populations.

## Teaser

Hybrid water frogs in the *Pelophylax esculentus* complex typically reproduce through hybridogenesis, a process that clonally transmits one parental genome while eliminating the other. Our study reveals that, despite this generally stable mechanism, hybrid males from multiple populations frequently exhibit atypical gametogenic patterns, including variable genome elimination and transmission of different parental genomes. These deviations, may be associated with genomic introgressions, highlighting the evolutionary flexibility and complexity of hybrid reproductive systems.

## Graphical abstract

**Figure:**
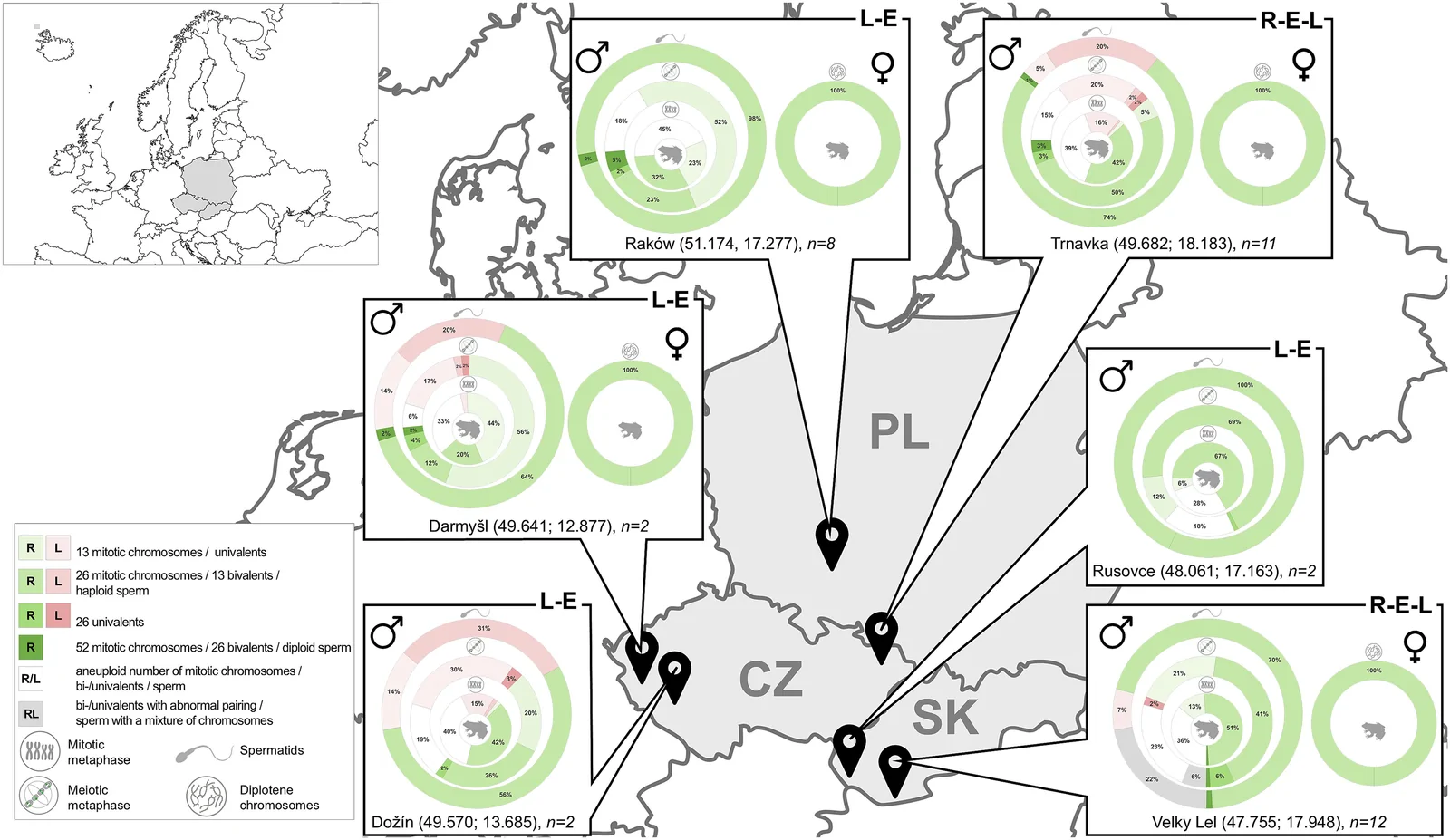

## Introduction

Ancient and ongoing hybridization is relatively widespread among animals and plants [1–3]. However, hybrids often exhibit lower viability and sterility caused by germ cell degeneration [4, 5] or improper pairing of orthologous chromosomes during meiosis [6, 7]. Nevertheless, gametogenesis in hybrids can be modified to avoid pairing between two diverged sets of parental chromosomes in meiosis, thus leading to a variety of clonal and hemiclonal reproductive modes, including parthenogenesis, gynogenesis, kleptogenesis, and hybridogenesis [2, 8, 9]. Some reproductive modes, such as parthenogenesis and gynogenesis, completely preserve heterozygosity of two species and ensure their clonal propagation without sexual species contribution [9–11]. Other reproductive modes, such as kleptogenesis and hybridogenesis, can fix only half of the parental species’ genome by clonal propagation but require crosses with sexual species to produce hybrids in each generation [2, 9, 12, 13]. Nevertheless, such hybrids may thrive by continuously refreshing part of their genome from co-existing sexual species [2, 9, 12]. Furthermore, hemiclonal hybrids often produce gametes with varying genomic makeup, fostering polyploid emergence and significantly increasing population complexity [9, 12, 14, 15]. A central, yet unresolved question is how stable genome elimination and transmission are across different hybrid population systems, geographic contexts, sexes, and whether genomic introgressions may affect asexual reproduction.

Hemiclonal reproduction through hybridogenesis, often accompanied by polyploidy, was found in European water frogs of the *Pelophylax esculentus* complex [12, 16, 17]. This complex includes parental species, *P. lessonae* (LL genotype) and *P. ridibundus* (RR genotype), which hybridize and produce hybrids, named *P. esculentus*. Hybrid frogs are represented by diploid (LR genotype), triploid (LLR, LRR), and, rarely, tetraploid forms [18, 19]. In diploid hybrids, one parental genome is typically eliminated, while the other is endoreplicated and transmitted to the offspring clonally [20–22]. In triploids, the genome present in a single copy is usually eliminated (L genome in LRR hybrids and R genome in LLR hybrids), while the remaining genomes participate in the formation of haploid [18, 23, 24] or diploid [19, 25] gametes. Nevertheless, fluctuation from canonical genome elimination and endoreplication pathways results in the production of different types of gametes, leading to variation in fertility of hybrid genotypes [23, 24, 26].

Across their range, *P. esculentus* cohabits with both parental species (so-called R-E-L system), only one of them (R-E or L-E system), or even without them (E system) [12, 16]. The L-E system is the most widespread and was found in France, Switzerland, Germany, Netherlands, Czech Republic, Slovakia, Poland, Hungary, Belarus, Latvia, Ukraine, and the European part of Russia [17, 27–29]. The wide distribution of the L-E system and regular propagation of *P. esculentus* in such system have been explained by the more effective elimination of *P. lessonae* genome and clonal propagation of *P. ridibundus* genome [28, 29] clearly recognizable even in F1 hybrids experimentally produced in laboratory conditions [30–32]. Results of artificial crossing experiments of *P. esculentus* from the L-E system with *P. lessonae* revealed mostly hybrid progeny, suggesting that such hybrids produced gametes with a *P. ridibundus* genome [27, 33, 34]. This pattern of hybridogenetic reproduction was also found in some R-E-L populations, where hybrids produced gametes carrying the *P. ridibundus* genome and backcrossed with *P. lessonae* to produce hybrids, with the *P. ridibundus* subpopulation not contributing to the hybrid reproduction [28, 31, 35]. A recent study of natural amplexus pairs from L-E system demonstrated the complexity of gamete types produced by hybrid females and males, including Rx and/or Lx or Lx, Ly and/or Rx genomic combinations (x and y indicates sex chromosomes), facilitating the restoration of both *P. esculentus* and *P. lessonae* genotypes [36]. Nevertheless, despite extensive studies of crosses and hybrids from the L-E system, which has generally been considered more stable than other population systems, detailed analyses of gametogenesis remain limited. Moreover, recent studies of eastern L-E and R-E-L systems suggest that gametogenesis may be more variable than previously assumed [28, 31, 37–39].

Another key assumption of clonal or hemiclonal reproduction is the effective isolation of parental genomes within hybrids, preventing their intermixing [40–44]. Although occasional rearrangements and introgressions between *P. lessonae* and *P. ridibundus* chromosomes were observed in diploid hybrids [37, 42] it is considered a rare event. Nevertheless, intensive genome introgressions from closely related species were found within the *P. ridibundus* genome due to historical or ongoing hybridization between *P. ridibundus* and closely related taxa, like *P. kurtmuelleri* (or *P. ridibundus kurtmuelleri* according to some authors; e.g. [45]) and *P.* cf *bedriagae* [39, 46–50]. Moreover, extensive exchanges between *P. lessonae* and *P. ridibundus* chromosomes, accompanied by potential genome introgressions from the additional *Pelophylax* species, were found in some hybrids from the R-E system in eastern Ukraine [38]. Although the role of these introgressions in the gametogenesis of *P. esculentus* is unclear, it was suggested that they might affect hybridogenetic reproduction [28, 38, 39].

Based on these observations, we hypothesize that genome elimination in hybrids from Central European L-E system is biased toward the consistent removal of the *P. lessonae* genome, predominantly resulting in gametes with *P. ridibundus* genome. Nevertheless, we suggest that this pattern is not completely stable and may be disrupted in some individuals. We further hypothesized that atypical gametogenic pathways are associated with chromosomal leakage or interspecific genomic introgressions. Based on these assumptions, we tested three predictions: (i) most diploid hybrids predominantly produce germ cells carrying the *P. ridibundus* genome; (ii) gametogenesis in both hybrid males and females is similar (iii); germ-cell populations and gametes with variable genome composition and ploidy occur if genome elimination and endoreplication are unstable; and (iv) individuals exhibiting the strongest gametogenic deviations may also show evidence of chromosomal substitutions or introgressed genomic regions.

To test our hypotheses, we investigated gametogenesis in hybrids of both sexes from multiple populations in Poland, Slovakia, and the Czech Republic, representing both L-E and R-E-L systems, that have never been analyzed so far. These populations were selected to represent different population structures, allowing us to compare whether typical genome elimination patterns are consistent across systems. The inclusion of multiple geographically distinct populations also enabled us to assess whether gametogenic variability is a local phenomenon or a recurrent feature across the Central European range of *P. esculentus*. Since cytogenetic methods provide the most reliable means of identifying ploidy and genome composition at different stages of gametogenesis in hybrids, we performed fluorescent *in situ* hybridization (FISH), to examine spermatogonia cells, spermatocytes, and spermatids in 26 hybrid males. Additionally, we analyzed chromosome composition of diplotene oocytes from 11 hybrid females from different localities. Finally, using comparative genomic hybridization (CGH), we revealed the large-scale genome introgressions within the genomes of the studied hybrid individuals and assessed its potential role in gametogenesis.

## Materials and methods

### Sample collection

We collected 37 hybrid individuals from four L-E and two R-E-L systems in Central Europe. Five males and three females were collected from Raków (51.173, 17.277), Poland; seven males and five females from Veľký Lél (47.755; 17.948), and two males from Rusovce (48.061; 17.163) in Slovakia; nine males and two females from Trnávka (49.682; 18.183), two males from Dožín (49.570; 13.685), and one male and one female from Darmyšl (49.641; 12.877) in the Czech Republic (Supplementary Table S1). All manipulations with animals were carried out under national and international guidelines. Techniques used in animal capture, tissue sampling, and euthanasia sought to minimize animal suffering. Each individual was anesthetized and subsequently euthanized by submersion in a 0.5% solution of 3-aminobenzoic acid ethyl ester (MS 222). Permission No. DZP-WG.6401.111.2021.TŁ obtained from the General Director of Environmental Protection, Poland; Animal collection in Slovakia was conducted under Permission No. 519/2022 6.3.

### Taxonomic evaluation of animals

Initially, the hybrids were identified based on a combination of morphological features as described by Kierzkowski et al. 2011 [51]. Polymerase chain reaction – restriction fragment length polymorphism (PCR-RFLP) method was further utilized to assess their genotypes [52]. PCR amplification of the recombination-activation gene 1 (*Rag1*) was performed using primers Mart-FL1 and AMP-R1 rana, and the resulting products were digested with the restriction enzyme DraII according to Cuevas et al. 2022 [52] and Doležálková-Kaštánková et al. 2024 [53]. Digested fragments were separated on a 1.5% agarose gel.

Genotypes of individuals were determined by amplification of alleles in 14 polymorphic microsatellite markers according to Doležálková-Kaštánková et al. 2024 [53]: Multiplex 1 (Res14, RICA18, RICA1b5, RICA2a34F, Ga1a19, RICA5, and Rrid013A) and Multiplex 2 (Re1Caga10, Res22, RICA1b6, Re2Caga3, Rrid059A, Rrid082A, and Rrid169A). These loci were widely used to distinguish Central-European water frogs’ taxa [23, 24, 27, 54]. Fragment-length analysis was utilized using an ABI 3730 Avant capillary sequencer (Applied Biosystems, Foster City, California, USA) with an internal size standard (GeneScan-500 LIZ, Thermo Fisher Scientific, Waltham, MA, USA). Allele identification and scoring were performed by GeneMapper v. 3.7 software (Applied Biosystems, Foster City, California, USA).

Finally, to confirm species identification, we utilized CGH and FISH with probes targeting species-specific tandem repeats *RrS1* [55] and *PlesSat01–48* [32] on mitotic chromosomes in all individuals.

### Preparation of metaphase and lampbrush chromosomes

Following euthanasia in 0.5% MS-222 (Sigma-Aldrich, St. Louis, MA, USA), intestines, ovaries (in females), and testes (in males) were dissected. All hybrid females (n = 11) were used for lampbrush chromosome analysis following Dedukh et al. 2015 [26]. Lampbrush chromosomes were extracted from oocyte nuclei following standard procedures described by Callan et al. 1987 [56], with modifications suggested by Gall et al. 1991 [57]. The resulting lampbrush chromosomal spreads were dehydrated in ethanol solutions (50%, 70%, 96%) and air-dried.

All hybrid males were used for the meiotic analysis following Doležálková-Kaštánková et al. 2024 [53] and Pustovalova et al. 2022 [58]. Adult individuals were injected with 1 ml of a 0.3% colchicine solution (Sigma-Aldrich) 24 h before euthanasia and dissection. After dissection, the testes were split into two parts. One part was fixed in 2% PFA in 1× PBS (Phosphate-buffered saline) for 1 h and used for subsequent analysis of gonadal microanatomy. Another part, along with the intestines, was utilized for cytogenetic analysis. To achieve this, the tissues were immersed in a hypotonic solution for 25 min and then transferred to a 3:1 fixative (3 volumes of ethanol: 1 volume of glacial acetic acid) for 30 min. Following two changes of the fixative, the tissues were stored at 4°C to prepare metaphase chromosome slides. A fragment of the fixed gonad tissue was suspended in 100 μl of a 70% acetic acid solution and disintegrated using two forceps. The resulting cell suspension was deposited onto slides preheated to 60°C. After the solution evaporated, metaphase spreads and interphase nuclei remained on the slide.

### Probe labeling and fluorescence *in situ* hybridization

The probes for FISH were labeled using PCR. Two kinds of probes were used for FISH:

(1) a biotin-labeled *RrS1* probe specific to the pericentromeric tandem repeat of *P. ridibundus* [55]. PCR was carried out with the genomic DNA of *P. ridibundus* and the following primers according to a previously published protocol [32] (Forward: 5′-AAGCCGATTTTAGACAAGATTGC-3′ and Reverse: 5′-GGCCTTTGGTTACCAAATGC-3′).

(2) a digoxigenin-labeled new *PlesSat01–48* probe specific to the two pairs of *P. lessonae* chromosomes [32]. PCR was carried out with the genomic DNA of *P. lessonae* and the following primers: Forward: 5′-TTTGGCTTCCAAGGGCCGGG-3 and Reverse: 5′-TGACCAAAAACGACACTCCC-3′.

FISH was conducted on mitotic and meiotic metaphase chromosomes following Choleva et al. 2026 [32]. Prior to FISH, slides were treated with 200 ng/ml RNase solution in 2× SSC (saline-sodium citrate buffer) for 1 h at room temperature (RT) and 0.1 mg/ml pepsin solution in 0.1 M HCl for 7 min at RT. Chromosomal DNA was denatured in a 75°C solution comprising 70% formamide and 2× SSC. Subsequently, slides were treated with a series of ice-cold (−20°C) ethanol (50%, 70%, and 96%). 10 ng/μl of PCR-labeled probes (5 ng/μl for the probe to *RrS1* and 5 ng/μl for the probe to *PlesSat01–48*) were mixed with a hybridization mixture consisting of 50% formamide, 10% dextran sulfate, 2× SSC (sodium chloride-sodium citrate buffer) and a 10–50-fold excess of salmon sperm DNA. Afterward, probes were denatured at 95°C for 10 min in the heating block (Grant Bio PCH1, UK). After denaturation, the probes were placed on an ice rack. The hybridization mixture was then applied to the slides, covered with coverslips, and sealed with rubber glue.

The slides were incubated for 12–24 h at RT. Following hybridization, slides underwent three washes in 0.2× SSC at 45°C with shaking. Biotin was identified using avidin conjugated with the Alexa488 fluorochrome (Invitrogen, Carlsbad, CA, USA), and digoxigenin was detected with anti-digoxigenin conjugated with the rhodamine fluorochrome (Invitrogen, USA). After washing in 4× SSC at 42°C, slides were dehydrated in an ethanol series (50%, 70%, and 96%), dried, and mounted in DABCO antifade solution containing 1 μg/ml DAPI (Vector, USA).

### Comparative genomic hybridization

Comparative genome hybridization (CGH) was performed following Pustovalova et al. 2025 [38], Bezlepkina et al. 2026 [39] and Doležálková et al. 2016 [59]. The total genomic DNA was extracted from the skeletal muscle of *P. lessonae* and *P. ridibundus* tissue using the DNeasy Blood and Tissue Kit (Qiagen, Hilden, Germany). Afterward, the genomic DNA samples were labeled with 2′-deoxyuridine, 5′-triphosphate (dUTP) combined with digoxigenin (for the *P. lessonae* genome, 1 μg per slide) and biotin (for the *P. ridibundus* genome, 1 μg per slide) through nick translation (Abbott, Chicago, USA). Nick translation was carried out at 17°C for 2.5 h. *P. ridibundus* and *P. lessonae* probes were then combined and mixed with salmon sperm DNA before precipitation. The mixture was then dissolved in a hybridization solution (50% formamide, 10% dextran sulfate, 2× SSC, 0.04 M NaPO_4_ [sodium phosphate] buffer, 0.1% sodium dodecyl sulfate, Denhardt’s reagent according to Pustovalova et al. 2025 [38], Dudzik et al. 2023 [44] and Doležálková et al. 2016 [59]). Probe and chromosome denaturation were performed similarly to the FISH experiments. For the hybridization of the probe and chromosomal DNA, slides were incubated for 3 days at 37°C. Subsequently, the slides were washed in 0.2× SSC at 60°C while shaking. Biotin-conjugated *P. ridibundus* probe was detected with the streptavidin conjugated with Alexa488 fluorochrome (Invitrogen, Carlsbad, CA, USA), and digoxigenin-conjugated *P. lessonae* probe was visualized using the anti-digoxigenin antibodies conjugated with rhodamine fluorochrome (Invitrogen, Carlsbad, CA, USA). Biotin and digoxigenin detection, along with subsequent washing and treatment, followed the procedures described for FISH.

### Fluorescence microscopy

Slides with chromosome spreads were analyzed by an Axio Imager Z2 microscope equipped with a CoolCube 1 black/white digital camera (MetaSystems, Altlussheim, Germany). The MetaSystems platform was utilized for automatic search, capture, and image processing. IKAROS and ISIS imaging software (MetaSystems) were also applied for capturing and the initial processing of the images. The preparation of the final figures was performed in Adobe Photoshop CS6.

### Scoring of male cytogenetic categories

Based on the stage of spermatogenesis, number of chromosomes and signals of *RrS1* and/or *PlesSat01–48*, the metaphases of each individual were applied to the following categories: (i) Meiotic metaphases: a minimum of 49 metaphase I figures were scored per frog (range 49–278). Cells were classified as: *P. ridibundus* (*RrS1*-positive) or *P. lessonae* (*PlesSat01–48* positive, two signals in haploid chromosomal composition) exclusively with 13 bivalents, 13 univalents, 26 bivalents, 26 univalents, 13 tetravalents, aneuploid (irregular chromosome number), or mixed *P. ridibundus* + *P. lessonae* (26 or 39 univalents). (ii) Spermatids: a minimum of 22 spermatids were scored per frog (range 22–128), classified as haploid with *P. ridibundus* genome composition (*RrS1*-positive, with signal numbers ranging from 6 to 13, *PlesSat01–48*-negative), haploid with *P. lessonae* genome composition (*RrS1*-negative/weak, *PlesSat01–48*-positive with two signals), diploid (*RrS1*-positive with 26 signals), or other/mixed (irregular signal number). (iii) spermatogonia cells: a minimum of three mitotic metaphases were scored per frog (range 3–66), classified as *P. ridibundus* or *P. lessonae*-only 13 chromosomes (haploid), 26 chromosomes (diploid), 39 chromosomes (triploid) or aneuploid. For a further statistical analysis all counts were converted to proportions (%) relative to the total scored cells per category per individual (Supplementary Table S3).

### Whole-mount fluorescence *in situ* hybridization and confocal microscopy

Whole-mount fluorescence *in situ* hybridization was conducted on testis fragments from adult individuals using the two species-specific probes according to Bezlepkina et al. 2026 [39] and Dedukh et al. 2020 [60]. Gonads, preliminarily fixed in a 2% paraformaldehyde solution in 1× PBS for 1.5 h, were stored at 4°C in 1× PBS with 0.02% sodium azide until usage. Before the whole-mount FISH, tissues were incubated in a 0.5% solution of Triton X-100 in 1× PBS for 4–5 h at RT, followed by a 15-min wash in 1× PBS. Subsequently, the tissues were impregnated with a solution comprising 50% formamide, 10% dextran sulfate, and 2× SSC for 3–4 h at 37°C. Afterward, tissues were transferred to the hybridization mixture (50% formamide, 2× SSC, 10% dextran sulfate, 20 ng/μl probe (10 ng/μl for probe to *RrS1*, 10 ng/μl for probe to *PlesSat01–48*), and a 10- to 50-fold excess of salmon sperm DNA/yeast DNA) and heated at 75°C for 10 min for DNA and probe denaturation. After hybridization for 24 h at RT, tissues were washed in 0.2× SSC solutions at 45°C for 3 times. Biotin was detected by incubation with streptavidin-Alexa488 (Invitrogen); digoxigenin was visualized by the anti-digoxigenin conjugated with rhodamine (Invitrogen). Finally, the tissues were stained with DAPI (1 μg/μl) (Sigma-Aldrich), prepared in 1× PBS at RT overnight.

Analysis of the 3D morphology of the gonads was performed by laser scanning confocal microscopy using a Leica TCS SP5 based on the inverted microscope Leica DMI 6000 CS (Leica Microsystems, Wetzlar, Germany). Tissues were placed in a drop of DABCO antifade solution containing 1 mg/ml DAPI. Specimens were analyzed in XYZ planes using HC PL APO 20×, 40× objectives. Diode, argon, and helium-neon lasers were used to excite the DAPI fluorescent dye and the Alexa488 and Cy3 fluorochromes, respectively. Images were obtained using LAS AF software (Leica Microsystems). The processing and counting of the number of micronuclei and the fluorescent signal in the cells were performed using the LAS AF software (Leica Microsystems).

### Statistical analysis

Non-parametric tests were used for univariate and bivariate analyses, as cytogenetic proportions deviated from normality (Shapiro–Wilk test, all *P* < 0.05). Between-population differences in individual-level proportions were assessed using the Kruskal-Wallis test, followed by appropriate pairwise comparisons where relevant. Two-group comparisons were performed using the Mann–Whitney U test (α = 0.05, two-tailed).

Differences in frequency distributions of pooled absolute counts were evaluated using Pearson’s chi-square (χ^2^) tests of independence, and effect size was quantified using Cramér’s V = √(χ^2^ / [n × (min(r,c) − 1)]).

Bivariate relationships among cytogenetic compartments were assessed using Spearman’s rank correlation coefficient (ρ) and were restricted to diploid individuals (n = 24) to avoid ploidy-associated non-independence; triploid individuals were excluded from this analysis.

Principal component analysis (PCA) was conducted on nine z-score-standardized cytogenetic variables (13 *P. ridibundus* meiotic bivalents, aneuploid *ridibundus* meiotic bivalents, *P. lessonae* meiotic/mitotic metaphases, haploid *ridibundus* spermatids, haploid *P. lessonae* spermatids, aneuploid spermatids, spermatogonia with 26 *ridibundus* mitotic chromosomes, aneuploid spermatogonia, and spermatogonia with *P. lessonae* mitotic chromosomes) using eigendecomposition of the correlation matrix. Variable contributions to each principal component were calculated as (loading^2^ / Σloading^2^) × 100.

Population centroids in principal component space were compared using Euclidean distances as a descriptive measure of multivariate separation among groups.

All statistical analyses were performed on male cytogenetic data only. As female gametogenesis was uniform across all individuals and populations, females were excluded from statistical comparisons (Supplementary Table S3–S5).

## Results

We analyzed gametogenesis in 26 adult hybrid males and 11 hybrid females from six different populations located in Poland, Slovakia, and the Czech Republic (Supplementary Table S1 and S2). Firstly, we applied FISH with species-specific probes to visualize the pericentromeric regions of all *P. ridibundus* chromosomes *(RrS1)* and two chromosomes of *P. lessonae (PlesSat01–48)* (Figure 1 and 2; Supplementary Figure S1). Furthermore, to investigate the presence of large-scale genome rearrangements and potential introgressions, we utilized CGH on somatic (n = 26) and testicular (n = 6) tissues of hybrid males (Figure 3). To assess the potential fertility of hybrid males, we analyzed the gonadal microanatomy of 18 hybrid individuals (Figure 4, Supplementary Figure S2). Finally, we analyzed genome composition and ploidy in diplotene oocytes from 11 diploid hybrid females (Supplementary Figure S3).

**Figure 1:**
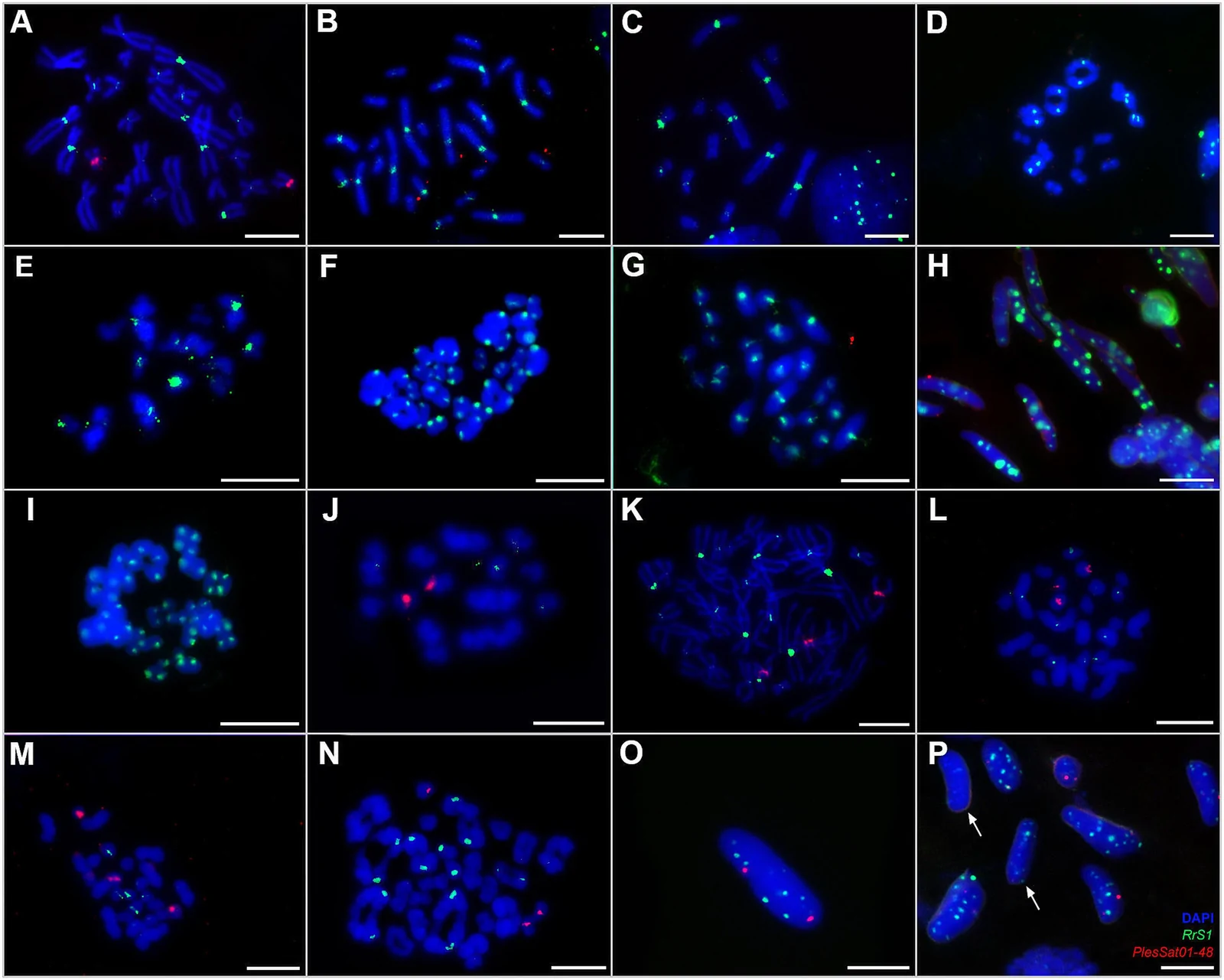
FISH-based identification of genotypes and chromosomal composition in mitotic and meiotic spreads of diploid (A–J) and triploid (K–P) hybrids from Slovakia. *P. ridibundus* chromosomes are identified by the *RrS1* probe (green) in the pericentromeric regions of all chromosomes, whereas *P. lessonae* chromosomes are identified by the lack of signals from *RrS1* probe and the presence of signals from *PlesSat01–48* probe (red) in the pericentromeric regions of two small chromosomes. Chromosomes are counterstained with DAPI (blue). Somatic chromosomal plate of a diploid hybrid with 13 *P. ridibundus* chromosomes and 13 *P. lessonae* chromosomes (A). Mitotic metaphases from testicular cell spreads with 26 (B) and 13 (C) *P. ridibundus* chromosomes. Spermatocytes with 13 bivalents (D), 13 univalents (E), 26 bivalents (F) and 26 univalents (G) including only *P. ridibundus* chromosomes. Haploid spermatids with *P. ridibundus* chromosomes (H). Spermatocytes with 13 tetravalents (I) including only *P. ridibundus* chromosomes. Spermatocytes with 13 univalents (J) with *P. lessonae* chromosomes. Somatic chromosomal plate of a triploid hybrid with 15 *P. ridibundus* chromosomes having a bright green *RrS1* signal and around 24 *P. lessonae* chromosomes with three signals from *PlesSat01–48* probe (K). Spermatocytes with 26 univalents (L, M) and a mixture of around 24 bivalents and univalents of *P. lessonae* chromosomes (N). Instead of four *P. lessonae* chromosomes detected by *PlesSat01–48* probe, we observed three *P. lessonae* chromosomes in L, M, N. Aneuploid spermatids from a triploid hybrid had from two (O) to zero (P, indicated by arrows) *P. lessonae* chromosomes detected by *PlesSat01–48* probe. Scale bar = 10 μm.

**Figure 2:**
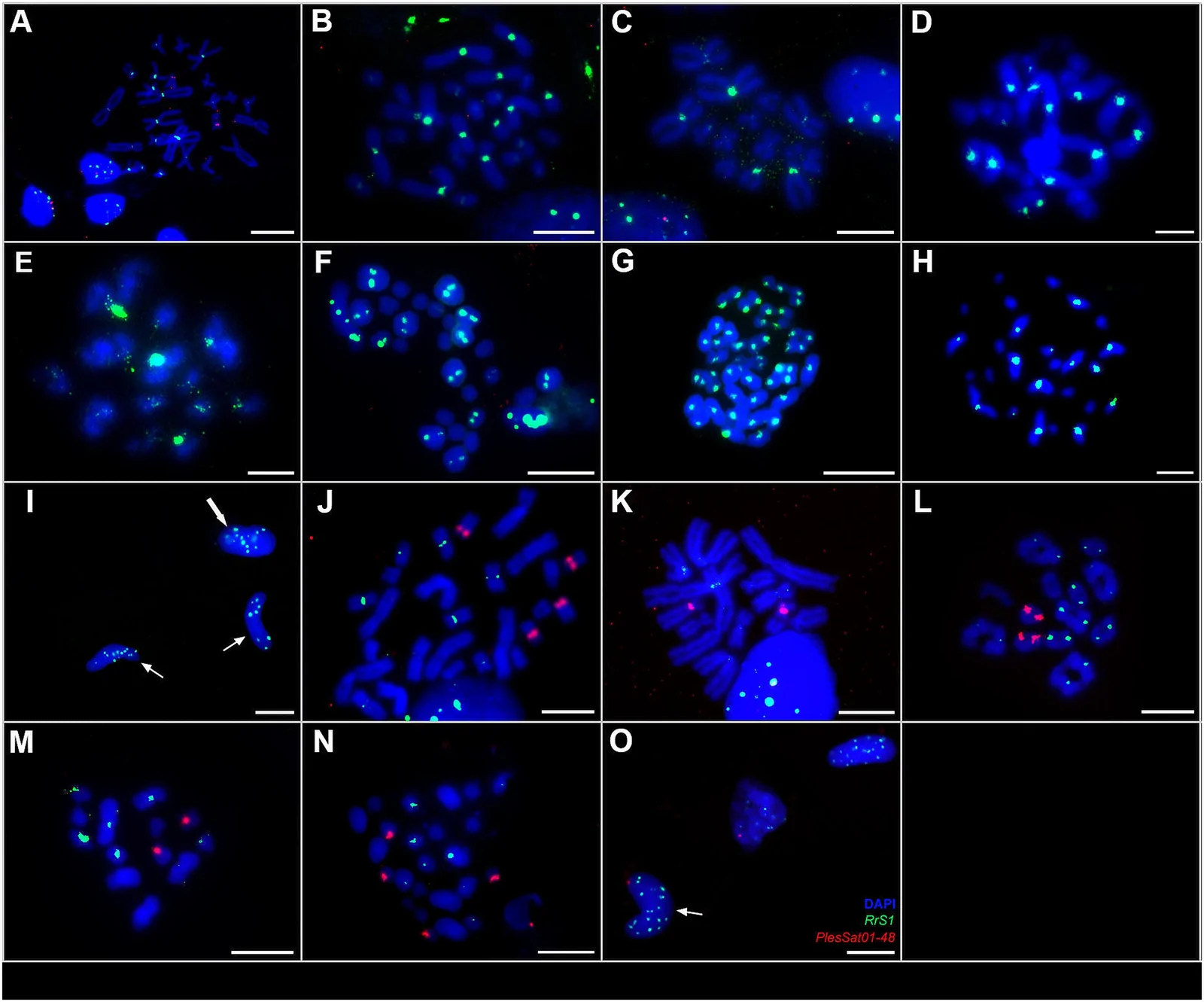
FISH-based identification of genotypes and ploidy in mitotic and meiotic spreads in diploid hybrids from the Czech Republic. *P. ridibundus* chromosomes are identified by the *RrS1* probe (green) in the pericentromeric regions of all chromosomes, whereas *P. lessonae* chromosomes are identified by the lack or decrease of signals from *RrS1* probe and the presence of signals from *PlesSat01–48* probe (red) in the pericentromeric regions of two small chromosomes. Chromosomes are counterstained with DAPI (blue). Somatic chromosomal plate with 13 *P. ridibundus* chromosomes and 13 *P. lessonae* chromosomes (A). Mitotic metaphases from testicular cell spreads with 26 (B) and 13 (C) *P. ridibundus* chromosomes. Spermatocytes with 13 bivalents (D), 13 univalents (E), 26 bivalents (F), 13 tetravalents (G) and 26 univalents (H) of *P. ridibundus*. A mixture of haploid (indicated by thin arrows) and diploid (indicated by thick arrow) spermatids (I) with *P. ridibundus* genomes. Mitotic metaphases from testis spreads with 26 (J) and 13 (K) *P. lessonae* chromosomes. Chromosomal spreads from spermatocytes with 13 bivalents (L), 13 univalents (M), and 26 univalents (N) of *P. lessonae* chromosomes. Spermatid with *P. ridibundus* and *P. lessonae* signals (indicated by arrow) (O). Scale bar = 10 μm.

**Figure 3:**
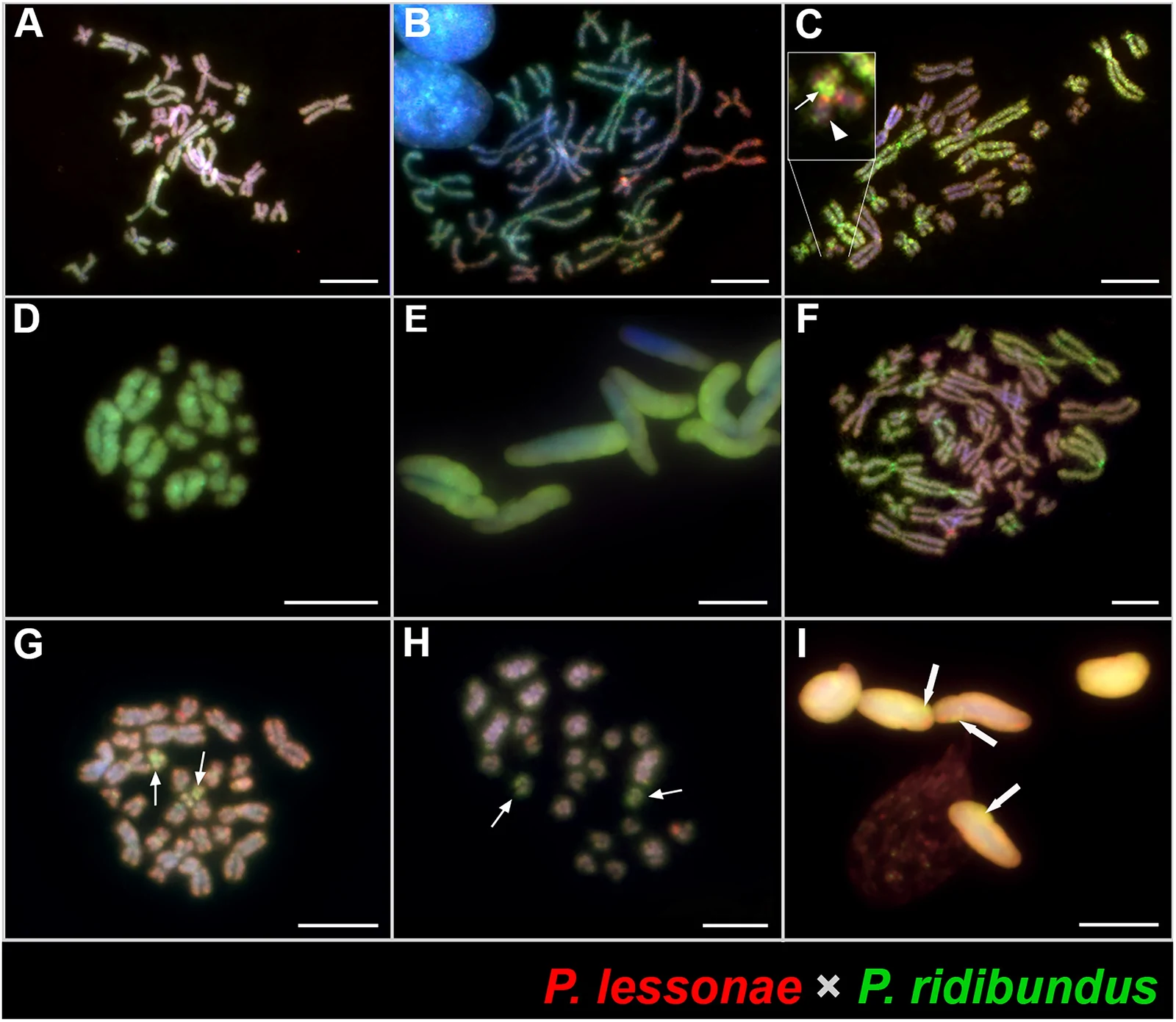
CGH on mitotic (A–C, F, G), meiotic (D, H) and spermatids (E, I) of diploid (A–E) and triploid hybrids (F–I). *P. ridibundus* chromosomes are shown in green; *P. lessonae* chromosomes are shown in red; DAPI is blue. Diploid hybrid somatic metaphase showing 13 *P. ridibundus* (green) and 13 *P. lessonae* (red) chromosomes in hybrids from Poland (A) and Czech Republic (B, C). In one diploid hybrid (C), a small acrocentric chromosome (enlarged in the inset) shows *P. ridibundus* signal in the pericentromeric region (shown by arrow in the inset) while the rest of the chromosome shows *P. lessonae* signals (shown by arrowhead in the inset). Arrows indicate an intense *P. ridibundus* signal in the pericentromeric region of presumably *P. lessonae* chromosomes. Spermatocytes from diploid hybrids with 13 bivalents of *P. ridibundus* chromosomes (D). Spermatids with signal corresponding to exclusively *P. ridibundus* chromosomes (E). Triploid hybrid somatic metaphase showing 15 *P. ridibundus* and 24 *P. lessonae* chromosomes (F). Mitotic chromosomal plate from testicular cell spreads of triploid hybrid had 26 chromosomes with 24 *P. lessonae* and two *P. ridibundus* chromosomes (indicated by thin arrows) (G). Spermatocyte spreads with 26 univalents from triploid hybrids included 24 *P. lessonae* chromosomes and two *P. ridibundus* chromosomes (indicated by thin arrows) (H). Spermatid with signals predominantly corresponding to *P. lessonae* chromosomes but also containing regions corresponding to *P. ridibundus* chromosomes (indicated by thick arrows) (F). Scale bar = 10 μm.

**Figure 4:**
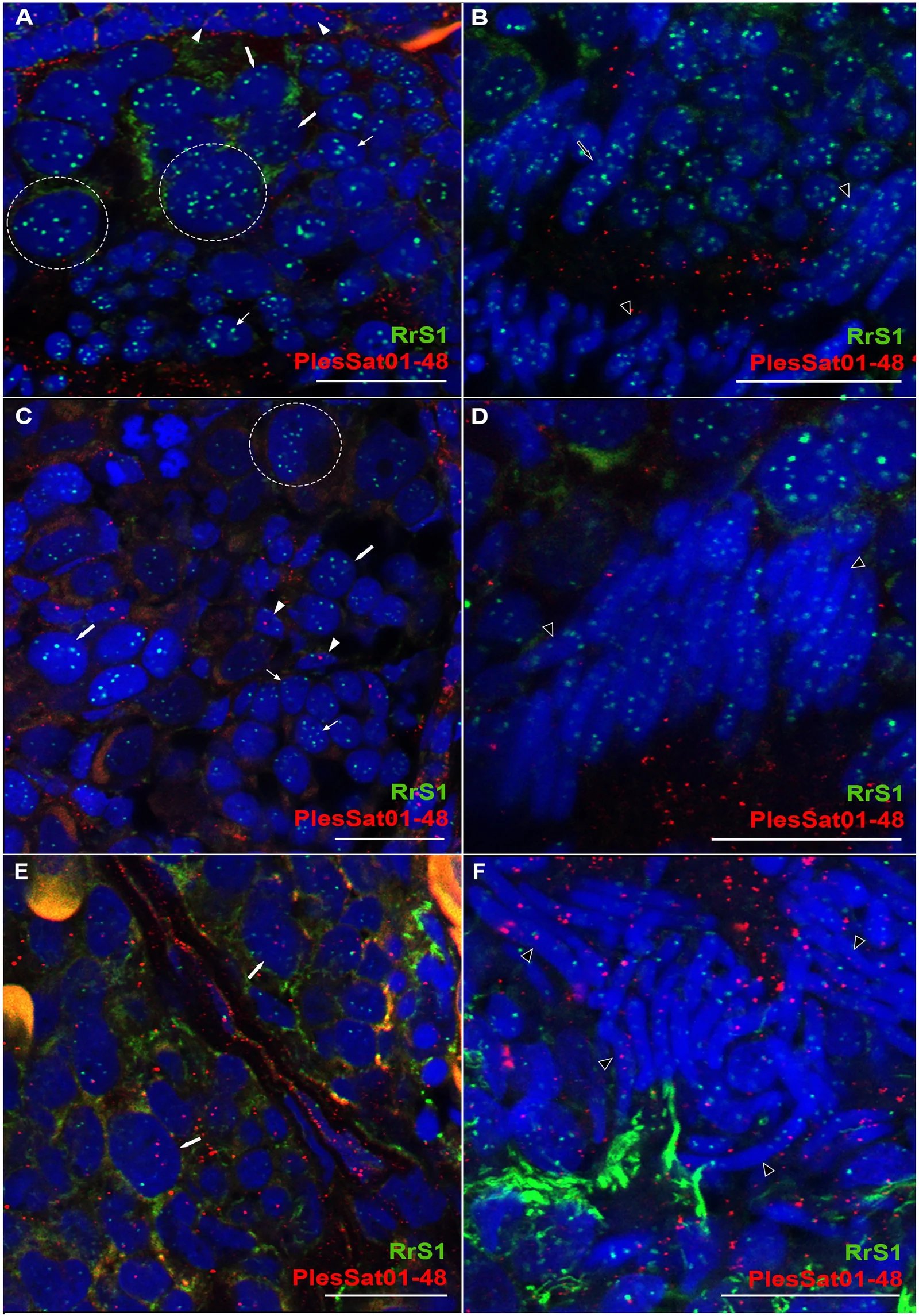
Whole mount FISH with *RrS1* (green) and *PlesSat01–48* (red) probes on gonadal fragments of diploid hybrids (A-D) from Poland and Slovakia, and triploid hybrids (E, F) from Slovakia. *RrS1* probe (green) marks *P. ridibundus* chromosomes; *PlesSat01–48* probe (red) marks two *P. lessonae* chromosomes. Chromatin is counterstained with DAPI (blue). In diploid hybrids from Poland (A, B) and Slovakia (C, D), spermatogonia included haploid (indicated by thin arrows), diploid (indicated by white thick arrows), and tetraploid (marked by dashed line) cells with exclusively *P. ridibundus* chromosomes; spermatids included haploid (pointed by black arrowheads) and diploid (shown by black thick arrow) *P. ridibundus* genomes. Somatic cells (pointed by white arrowheads) included both *P. ridibundus* and *P. lessonae* chromosomes. In triploid hybrids (E, F), spermatogonia included *P. ridibundus* chromosomes and *P. lessonae* chromosomes (pointed by white thick arrows). Spermatids included two signals of *P. lessonae* chromosomes and several signals of *P. ridibundus* varied in the intensity (pointed by black arrowheads in F). Scale bar = 25 μm.

### Genome composition and ploidy of hybrid individuals

FISH analysis of somatic tissues showed that all individuals from Poland and the Czech Republic, seven males and all females from Slovakia were diploid hybrids with 26 chromosomes and LR genome composition (Figure 1A, 2A; Supplementary Figure S1A; Supplementary Table S1). Two males from Slovakia were triploid, with 39 chromosomes, and presumably LLR genome composition (Figure 1K; Supplementary Table S1).

CGH analysis revealed no detectable large-scale genome rearrangements in diploid hybrids from Poland and Slovakia and in the majority of hybrids from the Czech Republic (Figure 3A, B). However, in three Czech males, one chromosome exhibited mixed species-specific signals, with *P. ridibundus*-specific signal in the short arm and *P. lessonae*-specific signal in the long arm (Figure 3C). Furthermore, in the two Slovak triploid males, CGH revealed 24 *P. lessonae* and 15 *P. ridibundus* chromosomes, deviating from the expected LLR configuration due to substitution of two *P. lessonae* chromosomes with *P. ridibundus* chromosomes (Figure 3F).

### Most hybrid males transmitted *P. ridibundus* genome in their germ cells

In most diploid hybrid males across all studied L-E and R-E-L populations (five males from Poland, seven males from Slovakia and seven males from the Czech Republic), spermatogenesis was characterized by germ cells carrying the *P. ridibundus* genome (Supplementary Table S2).

Mitotic analysis of testicular cell spreads revealed a significant difference in SSC composition among populations (χ^2^ = 27.49, df = 4, *P* < 0.001, Cramér’s V = 0.200). Diploid spermatogonia with 26 *P. ridibundus* chromosomes were the most frequent type (Poland: n = 24, 32%; Slovakia: n = 86, 52%; Czech Republic: n = 64, 29%) along with haploid spermatogonia with 13 *P. ridibundus* chromosomes (Poland: n = 17, 23%; Slovakia: n = 21, 13%; Czech Republic: n = 1, 0.5%) (Figure 1B and C;  2B and C; Supplementary Figure S1B and C). Additionally, we detected aneuploid cells (Poland: n = 33, 45%; Slovakia: n = 58, 35%; Czech Republic: n = 39, 38%) with *P. ridibundus* chromosomes (Supplementary Table S2).

Meiotic analysis showed a significant difference in the distribution of spermatocyte types among populations (χ^2^ = 315.99, df = 8, *P* < 0.001, Cramér’s V = 0.244). In 19 diploid hybrid males, spermatocytes predominantly formed 13 *P. ridibundus* bivalents (Poland: n = 359, 52%; Slovakia: n = 561, 50%; Czech Republic: n = 615, 38%) (Figure 1D;  2D;  3D; Supplementary Figure S1D). In addition, we observed spermatocytes with 13 *P. ridibundus* univalents (Poland: n = 162, 23%; Slovakia: n = 239, 21%; Czech Republic: n = 11, 0.7%), which may indicate either the metaphase of the second meiotic division following normal chromosome segregation or primary spermatocytes with 13 univalents (Figure 1E;  2E; Supplementary Figure S1E). We also found spermatocytes with 26 univalents (Poland: n = 34, 5%; Slovakia: n = 60, 5%; Czech Republic: n = 3, 0.1%), 26 bivalents and 13 tetravalents of *P. ridibundus* chromosomes (Poland: n = 11, 2%; Slovakia: n = 11, 1%; Czech Republic: n = 57, 3.5%) (Figure 1F and G;  2F–H; Supplementary Figure S1F and G). Also, we found aneuploid spermatocytes (Poland: n = 127, 18%; Slovakia: n = 249, 22%; Czech Republic: n = 147, 9%) with irregular chromosome compositions (Supplementary Table S2). Proportions of the aneuploid spermatocytes did not differ significantly between males from Poland and the Czech Republic (χ^2^ = 0.08, *P* = 0.782) or between Poland and Slovakia (χ^2^ = 3.74, *P* = 0.053), but were significantly more frequent in frogs from Slovakia than from the Czech Republic (χ^2^ = 5.93, *P* = 0.015).

Correspondingly, spermatids were mostly haploid and contained the *P. ridibundus* genome (Poland: n = 443, 98%; Slovakia: n = 538, 100%; Czech Republic: n = 671, 56%) (Figure 1H; 2I; 3E; Supplementary Figure S1H). However, diploid spermatids with *P. ridibundus* genome were also detected in hybrids from Poland (n = 9, 2%) and the Czech Republic (n = 9, 0.7%) (Figure 2I; Supplementary Figure S1I). The overall distribution of spermatid types differed significantly among populations (χ^2^ = 9.78, df = 2, *P* = 0.008), though the effect size was negligible (Cramér’s V = 0.077).

To further confirm the analysis of spreads from testes, we examined the gonadal microanatomy of intact gonadal tissues using species-specific probes to distinguish *P. ridibundus* and *P. lessonae* chromosomes in different types of germ-line cells. Spermatogonia, meiotic cells, and spermatids were identified morphologically following [39, 60–62]. In five hybrid males from Poland (Figure 4A and B), five diploid males from Slovakia (Figure 4C and D) and three diploid males from the Czech Republic, gonadal anatomy analysis confirmed the results from the testes spreads. Spermatogonia composition differed significantly among populations (χ^2^ = 27.61, df = 4, *P* < 0.001, Cramér’s V = 0.092). Most spermatogonia (Poland: n = 492, 64%; Slovakia n = 405, 66%; Czech Republic: n = 211, 25%) contained only 26 *P. ridibundus* chromosomes (Figure 4A, C; Supplementary Figure S2C and D). Some spermatogonia (Poland: n = 242, 31%; Slovakia: n = 124, 20%; Czech Republic: n = 92, 11%) had 13 *P. ridibundus* chromosomes, and individual spermatogonia (Poland: n = 34, 4%; Slovakia: n = 17, 3%) included around 52 *P. ridibundus* chromosomes (Figure 4A, C; Supplementary Table S2). The analysis of spermatids revealed significant differences among populations (χ^2^ = 18.42, df = 2, *P* < 0.001, Cramér’s V = 0.121), with the majority of spermatids (Poland: n = 465, 93%; Slovakia: n = 476, 95%; Czech Republic: n = 254, 39%) carrying 13 *P. ridibundus* chromosomes and individual spermatids (Poland: n = 35, 7%; Slovakia: n = 24, 5%) with around 26 *P. ridibundus* chromosomes (Figure 4B, D).

### Some diploid hybrid males transmitted preferentially *P. lessonae* genome

In five diploid hybrid males from the Czech Republic representing two L-E and one R-E-L systems, we detected germ cells which mostly carried *P. lessonae* genome (Supplementary Table S2).

Mitotic analysis of testicular cell spreads revealed predominantly haploid spermatogonia with 13 *P. lessonae* chromosomes (n = 26, 12%) and diploid spermatogonia with 26 *P. lessonae* chromosomes (n = 2, 1%) (Figure 2J and K). Moreover, in three out of five such hybrid males, we detected haploid spermatogonia with 13 *P. ridibundus* chromosomes (n = 32, 15%) and diploid spermatogonia with 26 *P. ridibundus* chromosomes (n = 19, 9%). The proportion of *P. lessonae* spermatogonia was positively correlated with the proportion of *P. lessonae* haploid spermatids across individuals (Spearman r = 0.818, *P* = 0.025, n = 5).

Meiotic analysis showed spermatocytes with 13 *P. lessonae* univalents (n = 362, 23%) and, less frequently, spermatocytes with 13 *P. lessonae* bivalents (n = 16, 1%) and spermatocytes (n = 22, 1%) with 26 *P. lessonae* univalent (Figure 2L–N). These males also produced spermatocytes with 13 *P. ridibundus* univalents (n = 216, 13%), spermatocytes with 26 *P. ridibundus* univalents (n = 21, 1%), spermatocytes with 13 *P. ridibundus* bivalents (n = 25, 1.5%), and occasional spermatocytes with 26 *P. ridibundus* bivalents (n = 2, 0.1%). The proportion of *P. lessonae* chromosomes in meiotic metaphases was positively correlated with *P. lessonae* haploid spermatids output (Spearman r = 0.937, *P* = 0.002, n = 5).

In these five Czech males, spermatids showed various genome compositions and ploidy, including haploid cells with the *P. ridibundus* genome (n = 266, 22%), haploid cells with the *P. lessonae* genome (n = 164, 14%), diploid cells with *P. ridibundus* genome (n = 4, 0.3%), and aneuploid cells (n = 90, 7%) (Figure 2O). The proportion of *P. ridibundus* haploid spermatids (mean 44.4% ± 18.3 SD) was significantly lower than in males, where *P. ridibundus* haploid spermatids constituted a mean of 90.6% of all scored spermatids (Mann–Whitney U = 58.0, *P* = 0.017, r = −0.84). Aneuploid spermatids were significantly more frequent in these five males compared to those which transmitted *P. ridibundus* genome exclusively (mean 18.0% ± 3.6 SD; U = 1.0, *P* = 0.0002, r = 0.97).

Gonadal microanatomy analysis performed for four hybrid males from the Czech Republic confirmed the presence of spermatogonia and spermatids with both parental genomes in different germ cell populations. We found diploid spermatogonia with *P. ridibundus* genomes (n = 28, 3%), diploid spermatogonia with *P. lessonae* genomes (n = 122, 14%), haploid spermatogonia with a *P. lessonae* genome (n = 260, 31%), and spermatogonia with a haploid *P. ridibundus* genome (n = 136, 16%) (Supplementary Figure S2A and B). Spermatids were predominantly haploid, carrying either *P. lessonae* (n = 246, 38%) or *P. ridibundus* (n = 136, 21%) genomes, with a small fraction of diploid spermatids containing *P. lessonae* genome (n = 18, 3%) (Supplementary Figure S2B; Supplementary Table S2). The distribution of cell categories differed significantly among individuals (χ^2^ = 130.9, df = 20, *P* < 0.0001, Cramér’s V = 0.506).

### Gametogenesis in triploid hybrid males

Mitotic analysis of testicular cell spreads from two triploid hybrid males from Slovakia revealed spermatogonia with 39 chromosomes (n = 9, 50%), 26 chromosomes (n = 7, 39%), and aneuploid chromosome composition (n = 11, 61%). CGH analysis showed that cells with 26 chromosomes consisted of 24 *P. lessonae* and 2 *P. ridibundus* chromosomes (Figure 3G).

Meiotic analysis revealed spermatocytes with 26 univalents containing both parental genomes (n = 29, 30%), spermatocytes with 39 chromosomes of both parental genomes (n = 17, 17%), and spermatocytes with 26 *P. lessonae* univalents (n = 20, 20%) (Figure 1L–N, 3G and H). Both the proportion of 13 *P. ridibundus* bivalents and the frequency of mixed-genome metaphases differed significantly from those in diploid males. Spermatids were predominantly diploid (n = 113, 75%) or aneuploid (n = 37, 25%) and contained contributions from both parental genomes (Figure 1O and P, 3I). The distribution of spermatids categories in triploid males differed significantly from diploid ones (χ^2^ = 2323.0, df = 8, *P* < 2.2 × 10^−16^, Cramér’s V = 0.704).

Gonadal microanatomy of one triploid hybrid male revealed spermatogonia with 1–3 signals of *P. lessonae* probe and 2–5 signals of *P. ridibundus* chromosomes, suggesting either aneuploidy or the leakage of *P. ridibundus* chromosomes replacing the corresponding *P. lessonae* ones. (Figure 4E). The analysis of the genome composition of spermatids in the triploid hybrid also showed the presence of both genomes (Figure 4F). We did not observe the substantial decreased number of spermatids on intact gonadal fragments compared to other males.

### Cytogenetic parameters do not differ between L-E and R-E-L systems

Meiotic metaphase composition, spermatid composition, and spermatogonia composition were compared among males from Poland (n = 5), Slovakia (n = 9), and Czechia (n = 12), as well as between males from L-E (n = 10) and R-E-L (n = 16) systems. No significant differences among geographical populations were detected for any meiotic variable, including the proportion of 13 *P. ridibundus* bivalents (Kruskal-Wallis H = 0.215, *P* = 0.898), aneuploid *P. ridibundus* metaphases (H = 2.086, *P* = 0.352), and *P. lessonae* chromosomes in meiosis (all *P* > 0.35). Similarly, no significant population-level differences were detected for any spermatid variable, including the proportion of *P. ridibundus* haploid spermatids (H = 0.885, *P* = 0.642), or for spermatogonia cells (H = 1.530, *P* = 0.465). Comparison between L-E and R-E-L system types revealed no significant differences in the proportions of haploid, aneuploid, or diploid spermatids (all *P* > 0.22) or spermatogonia cells (χ^2^ = 1.55, df = 2, *P* = 0.460, Cramér’s V = 0.062). Although the overall distributions of meiotic metaphase types (13 *P. ridibundus* bivalents, *P. ridibundus* univalents, aneuploid and mixed-genome metaphases) and spermatid types (*P. ridibundus* haploid, *P. lessonae* haploid, aneuploid, diploid, and mixed-genome) differed significantly between L-E and R-E-L males (χ^2^ = 163.96, *P* < 0.001 and χ^2^ = 77.19, *P* < 0.001, respectively). This was attributable to the unequal representation of genotypes across population types, specifically, the exclusive occurrence of triploid males in R-E-L system and the predominance of males with chromosomal rearrangements in L-E system, rather than to any direct effect of system type on gametogenesis.

Principal component analysis of 13 cytogenetic variables identified three components with eigenvalues greater than 1.0, together explaining 82.7% of total variance (PC1: 52.8%; PC2: 19.4%; PC3: 10.5%). PC1 separated frogs transmitting *P. ridibundus* genome (positive scores) from those retaining and transmitting *P. lessonae* genome (negative scores). PC2 was driven by mixed-genome meiotic metaphases and mixed-genome spermatids, separating triploid individuals from all diploid males. In PC1-PC2 space, the three genotype groups occupied distinct regions: diploid frogs with a karyotype without chromosomal rearrangements clustered at positive PC1 scores, diploid frogs with a karyotype with chromosomal rearrangements at intermediate negative scores, and triploid frogs were displaced along positive PC2. Population of origin and system type showed overlapping distributions on both axes (Supplementary Figure S4).

### Oogenesis in diploid hybrid females

Analysis of 162 diplotene oocytes from all diploid hybrid females (three from Poland, five from Slovakia, three from the Czech Republic) revealed only 13 bivalents corresponding to the *P. ridibundus* genome. FISH with both species-specific probes confirmed the presence of *P. ridibundus* chromosomes (Supplementary Figure S3), suggesting canonical gametogenesis and ability to form functional eggs with haploid *P. ridibundus* genome. No univalents or abnormalities in chromosome pairing were detected.

## Discussion

### Dominance of the canonical hybridogenesis pathway in diploid hybrids from Central European populations

A central question of this study was whether hybrid populations from Central Europe follow a uniform gametogenic mechanism. Across 26 hybrid males and 11 hybrid females from four L-E and two R-E-L systems, our data consistently support the dominance of the canonical hybridogenetic pathway, which involves premeiotic elimination of the *P. lessonae* genome and clonal transmission of the *P. ridibundus* genome to the gametes. Principal component analysis indicates that the key features of canonical hybridogenesis do not vary independently but instead form a tightly coordinated system. Variables related to *P. ridibundus* chromosome pairing, haploid sperm production, and spermatogonia composition align along a single dominant axis of variation, suggesting that these processes are functionally integrated and governed by a common underlying mechanism. Patterns observed in males and females from Poland, Slovakia, and the Czech Republic are consistent with previous studies of hybrids from L-E and R-E-L systems [22, 27, 28, 31, 62–67].

The analysis of gonadal microanatomy in adult hybrid males did not reveal the presence of micronuclei, which serve as a marker for genome elimination in hybrid tadpoles [31, 60–62, 68, 69], suggesting that the genome composition of spermatogonia in adult hybrid males is established during early embryogenesis in pregametogenesis, and does not occur during the gametogenesis of adult hybrids.

### Alternative gametogenic pathways were found in males but not in females

Despite the dominance of the canonical pathway, our data demonstrate that alteration of gametogenesis occurs repeatedly in males but not in females from various localities. Nearly half of the analyzed diploid males from L-E and R-E-L systems in the Czech Republic had spermatogonia with 26 *P. lessonae* chromosomes and spermatocytes with 13 bivalents formed by *P. lessonae* chromosomes. Accordingly, these males produced mostly spermatids with *P. lessonae* genomes. These results indicate the premeiotic elimination of the *P. ridibundus* genome, while *P. lessonae* genome undergoes endoreplication and transmission to the progeny. Notably, hybrid males producing gametes with the *P. lessonae* genome are crucial for the maintenance of the R-E system [53, 70, 71] and were occasionally found in hybrids from the E system [23, 72]. In most L-E and R-E-L systems, hybrids producing *P. lessonae* gametes have not been documented [22, 27, 28, 31, 62–64]. Although, we did not observe spermatogonia and spermatids representing *P. lessonae* genome in the L-E system from studied Polish locality, earlier gametes with the *P. lessonae* genome were shown for hybrids from this locality based on the crossing experiment results [36]. At a nearby locality within the L-E population, we previously identified diploid hybrid males whose spermatogonia, spermatocytes, and spermatids contained *P. ridibundus* or *P. lessonae* genomes [62].

Moreover, in our study, several males simultaneously produced germ cells of both genomic types within the same gonad, generating a mix of haploid *P. ridibundus* and haploid *P. lessonae* gametes. This phenomenon, known as amphispermy [73] or amphigameticity [58], appears to be a distinctive feature of Central and Eastern European R-E systems [38, 53, 54, 58, 59, 70, 73, 74]. The high frequency of amphispermic males observed in some Czech localities suggests that the underlying gametogenic mechanism may be shared between the studied L-E/R-E-L and R-E systems, despite differences in population structure. The production of both *P. ridibundus* and *P. lessonae* gametes has important consequences for gene flow between clonally transmitted genomes, particularly in R-E-L system, where hybrids can reproduce with both parental species. Crosses with different parental species allows clonally transmitted genomes to recombine after reintroduction into corresponding sexual species, thereby reducing the accumulation of deleterious mutations [75]. In contrast, in L-E system, the *P. ridibundus* genome is transmitted clonally in a subset of gametes, whereas gametes carrying the *P. lessonae* genome give rise to *P. lessonae* individuals.

A second major source of variability in diploid hybrid males involves the probable formation of diploid gametes in addition to haploid ones. Cytological configurations (26 bivalents or 13 tetravalents) indicate the presence of two rounds of premeiotic genome endoreplication of the retained genome following the genome elimination stage. Similar gametogenic alterations were observed in diploid hybrid males and females from the R-E system in eastern Ukraine [26, 38, 58], and were occasionally detected in diploid and triploid hybrid males from the R-E-L and E systems [62] but very rarely in hybrids from the L-E system [25, 62, 67]. After meiosis, such spermatocytes are expected to produce diploid spermatozoa. Although, we cannot determine whether the observed spermatids are aneuploid products of aberrant meiosis, the presence of tetraploid spermatogonia and spermatocytes (with 26 bivalents or 13 tetravalents), together with spermatids showing increased signal numbers, suggests that regular meiosis may occur, resulting in the production of functional diploid sperm. Diploid sperm are often considered less competitive through decreased motility [76–78], however it may play a key role in generating triploids and maintaining complex population systems [23, 25, 29].

We also detected abnormalities in genome elimination and endoreplication in hybrid males. In some hybrid males from all studied localities, we observed haploid gonial cells with only 13 chromosomes and corresponding haploid spermatocytes with 13 univalents. These results suggest premeiotic genome elimination of one parental genome without endoreplication of the remaining one, leading to haploid spermatogonia with only *P. ridibundus* and *P. lessonae* chromosomes correspondingly. Since we observed no evidence for genome elimination or micronuclei formation, we propose that at least some gonial cells remain haploid in adult hybrid males. Similar gametogenic alteration was observed in hybrid males and females from R-E, L-E, and R-E-L systems [38, 53, 58, 62, 70], but the massive occurrence of haploid premeiotic genome in germ cells was recently showed for diploid hybrid males from the eastern European L-E systems [39].

Further, in diploid hybrid males, we detected spermatocytes with 26 univalents (13 *P. ridibundus* and 13 *P. lessonae* chromosomes), indicating a failure of selective genome elimination and endoreplication. Similar configurations have been reported in the R-E system [26, 58, 70, 71]. In some males, spermatocytes with 26 univalents of either parental genome were also observed, suggesting that hybridogenetic processes occurred but chromosome pairing failed during meiosis. Such cells are unlikely to complete meiosis and contribute to functional gametes. These abnormalities have been associated with reduced fertility in R-E system [38, 53, 58] and were suggested to reduce fertility in hybrids in Eastern Ukraine and Central Europe [59, 70], although in some L-E and R-E-L systems, affected males remained fertile despite altered testis morphology [62]. Notably, in our study, we did not observe a reduction in spermatid production or other germ cell populations.

Together, our results reveal a pronounced sex-specific asymmetry in hybrid gametogenesis. While females consistently follow the canonical elimination of the *P. lessonae* genome, males exhibit extensive variability in genome elimination and endoreplication, generating a broad spectrum of germ cell ploidies and genome compositions within individuals. This difference likely reflects stronger selection against aberrant germ cells during oogenesis, whereas spermatogenesis is more permissive. Although abnormal oocytes have been reported in hybrids from L-E and R-E systems, such alterations are generally less frequent in females than in males from the same populations [26, 31, 58, 65, 70, 71]. The higher mechanistic flexibility observed in hybrid males enables the production of both haploid and diploid gametes and likely contributes to the maintenance of complex population systems, including the formation and persistence of triploids.

### The potential impact of chromosomal rearrangements and genomic leakage on hybrid gametogenesis

A key question addressed in this study is whether genomic instability contributes to variability in gametogenesis. The comparative analysis of gametogenesis in hybrids suggests differences in genome elimination and endoreplication among diploid hybrids from the different localities. The most variable gametogenic alterations were observed in hybrids from the Czech Republic, where both *P. lessonae* and *P. ridibundus* genomes are eliminated in different gonial cells of hybrids. However, this geographic pattern was not statistically supported at the individual level: meiotic composition and spermatid output did not differ significantly among populations (Kruskal-Wallis, all *P* > 0.14). This is further supported by comparison with R-E system in eastern Ukraine [38], where males from geographically and ecologically distinct localities also exhibited substantial variability in gametogenesis. In both datasets, canonical genome transmission coexists with aberrant outcomes, including amphispermy and aneuploid gamete formation. Despite some differences, both the Czech and Ukrainian systems show a common pattern in which gametogenic variability is closely associated with genomic irregularities rather than geographic origin.

To further investigate potential factors influencing hybrid gametogenesis, we analyzed the presence of large-scale genome rearrangements and potential introgressions using CGH. We did not find any large-scale introgressions in diploid hybrid frogs from Poland and Slovakia and most hybrids from the Czech Republic. Nevertheless, in three out of four amphispermic hybrid males, we observed one small acrocentric chromosome with a chimeric signal: a bright *P. ridibundus* signal in the pericentromeric region on a *P. lessonae* chromosome. Consistently, FISH with *RrS1* probes revealed stronger signals on *P. lessonae* chromosomes in Czech hybrids than in hybrids from other localities (this study and [31, 38, 53, 58, 60, 62, 67, 79]). Although *RrS1* is present in both parental species, it typically occurs at higher copy numbers in *P. ridibundus* [55]. Notably, an increased abundance of *RrS1* repeats in species from *lessonae* clade species has been proposed as a factor preventing genome elimination in hybrids with *P. ridibundus* [80, 81]. We therefore speculate that the elevated *RrS1* signal on *P. lessonae* chromosomes in these hybrids may interfere with their normal elimination, potentially contributing to variability in gametogenic pathways in Czech populations.

In this aspect, the repeated occurrence of this chimeric chromosome in hybrids with variable gametogenesis suggests that such structural variants may also interfere with the elimination of *P. lessonae* chromosomes. The multivariate position after PCA demonstrates that males with chromosomal rearrangements clearly separated from all other diploid hybrids. However, our data does not allow us to determine whether this chromosome has a causal role or is simply correlated with gametogenic alterations in these individuals. In the adjacent R-E system located in the Odra River basin, diploid hybrid males producing two types of gametes exhibit only weak differences between *P. lessonae* and *P. ridibundus* genomes when analyzed by CGH suggesting high genome intermixtures [59]. Furthermore, hybrids from the Odra River basin share clonal genome of *P. lessonae*, suggesting their common origin [54]. Although CGH in the current study did not reveal widespread genome restructuring, we cannot exclude the presence of smaller-scale rearrangements or exchanges between chromosomes within *P. ridibundus* and *P. lessonae* genomes. Such changes may remain undetected with the resolution of our approach.

An even more striking case of the whole chromosome introgression was found in two triploid hybrid males from Slovakia. In these males, we found 24 *P. lessonae* and 15 *P. ridibundus* chromosomes, suggesting “leakage” of two *P. ridibundus* chromosomes into the *P. lessonae* chromosome sets, where they replaced two *P. lessonae* chromosomes. Although earlier occasional genome rearrangements between *P. lessonae* and *P. ridibundus* chromosomes were found [37, 39, 42], whole chromosomal replacement has not been shown so far. Such chromosomes may result from errors during genome elimination, whereby two *P. lessonae* chromosomes are eliminated instead of *P. ridibundus* chromosomes that subsequently escape elimination. Alternatively, these triploid hybrids may derive from hybrid individuals exhibiting incomplete elimination of the *P. ridibundus* genome, leading to the formation of aneuploid gametes frequently reported in different hybrid systems [38, 53, 59, 62].

The analysis of gametogenesis in the triploid males from Slovakia revealed a relatively high number of spermatogonial stem cells and spermatocytes retaining the original ploidy, suggesting the absence of genome elimination in these cells. Such spermatocytes progress through meiosis and form spermatids that are classified as diploid and contain a mixture of both parental genomes. PCA indicates that gametogenesis in triploid males represents a distinct reproductive mode compared to that of diploid males with chromosomal rearrangements. The pattern observed in triploids is also distinct from the genomic instability described for hybrids from Ukrainian R-E system with intergenomic rearrangements [38]. In those hybrids, chromosomal regions of the third-species origin (*P.* cf. *bedriagae*) were incorporated into parental chromosomes, also abolishing genome elimination. However, Ukrainian males with rearrangements showed partial bivalent-like pairing between *P. ridibundus* and *P. lessonae* chromosomes during meiosis and produced at least some identifiable spermatids [38]. In contrast, the triploid males in the present study showed a complete absence of 13 bivalents and a total failure of haploid gamete production, suggesting a more severe disruption attributable to whole-chromosome rather than partial chromosomal introgression.

Although studied triploid hybrids exhibit a high number of gametogenic alterations, they produced spermatogonia and spermatocytes with 24 *P. lessonae* and 2 *P. ridibundus* chromosomes. This suggests that at least in some spermatocytes, the *P. ridibundus* chromosomal set was eliminated. Interestingly, the two *P. ridibundus* chromosomes avoid elimination and are retained together with the *P. lessonae* chromosomal sets. After meiotic completion, such cells result in haploid sperm with 11 *P. lessonae* chromosomes and two *P. ridibundus* chromosomes, observed at least in some spermatids.

Taken together, structural genomic variation, including chromosomal rearrangements and introgressions, may disrupt the recognition of chromosomes destined for elimination, as suggested by the association between chimeric chromosomes and altered gametogenesis observed here. At the population level, historical and ongoing hybridization events observed within *Pelophylax* genus [46, 49, 81] may promote the accumulation or fixation of such structural variants, thereby increasing the frequency of aberrant gametogenesis. These structural variants may contribute to the resistance of the *P. lessonae* genome to elimination potentially through differences in centromeric and pericentromeric repeat composition that affect kinetochore stability and chromosome segregation. Alternatively, the ability of the *P. ridibundus* genome to trigger *P. lessonae* genome elimination, including its preferential transmission potentially linked to “stronger” kinetochore properties, may be affected by historical introgressions or interchromosomal exchanges involving other *Pelophylax* species that do not normally undergo genome elimination. However, these scenarios remain speculative, as our data do not allow direct testing of their mechanistic roles. In addition, our results support a clear sex-specific component in genome elimination dynamics, with females exhibiting more stable gametogenesis compared to males, which display greater variability in elimination and endoreplication processes. This suggests that genome elimination in hybrids is likely shaped by an interplay between chromosomal structure and sex-specific regulation of gametogenesis.

## Supplementary Material

Supplementary_material_ioag171

## Data Availability

The data supporting the findings of this study are openly available within the manuscript and its supplementary materials.
