# Supplementary material for: Unexpected variability of gametogenic pathways and interspecific chromosomal leakage in Pelophylax esculentus hybrids
Source: Biol Reprod. 2026 Aug 19;115(4):1315–30. doi: 10.1093/biolre/ioag171 (PMC13669405; doi:10.1093/biolre/ioag171)
Supplement: Supplementary file 1 [file supplementary_material_ioag171.zip › Supplementary_Figures_S1-S4_ioag171.docx]

**Supplementary Materials**

**Unexpected variability of gametogenic pathways and interspecific chromosomal leakage in *Pelophylax esculentus* hybrids**

Martyna Fratczak, Veronika Labajova, Eleonora Pustovalova, Zuzana Majtánová, Krzysztof Kolenda, Petr Papežík, Marie Doležálková-Kaštánková, Magdalena Chmielewska, Lukáš Choleva, Peter Mikulíček, Dmitrij Dedukh

**Supplementary Figure S1. FISH-based identification of genotypes and ploidy in mitotic and meiotic spreads in diploid hybrids from L-E systems from Poland.** *P. ridibundus* chromosomes are identified by the *RrS1* probe (green) in the pericentromeric regions of all chromosomes, whereas *P. lessonae* chromosomes are identified by the lack of signals from *RrS1* probe and the presence of signals from *PlesSat01-48* probe (red) in the pericentromeric regions of two small chromosomes. Chromosomes are counterstained with DAPI (blue). Somatic chromosomal plate of diploid hybrid with 13 *P. ridibundus* and 13 *P. lessonae* chromosomes (A). Mitotic metaphases from testicular cell spreads with 26 (B) and 13 (C) *P. ridibundus* chromosomes. Chromosomal spreads from spermatocytes with 13 bivalents (D), 13 univalents (E), 26 bivalents (F), 26 univalents (G) of *P. ridibundus*. Haploid (H, pointed by thin arrows) and diploid (I, pointed by thick arrow) spermatids with *P. ridibundus* chromosomes. Scale bar = 10 µm.

**Supplementary Figure S2. Whole mount FISH with *RrS1* (green) and *PlesSat01-48* (red) probes on gonadal fragments of diploid hybrids from the Czech Republic.** *RrS1* probe (green) marks *P. ridibundus* chromosomes; *PlesSat01-48* probe (red) marks two *P. lessonae* chromosomes. Chromatin is counterstained with DAPI (blue). In one hybrid (A, B), spermatogonia included haploid (indicated by thin arrows), diploid (indicated by white thick arrows), and tetraploid (marked by dashed line) cells with exclusively *P. lessonae* chromosomes. Spermatids were haploid (labeled with black arrowheads) and diploid (labeled with black thick arrow) with exclusively *P. lessonae* chromosomes. In another hybrid (C, D), spermatogonia included haploid (indicated by thin arrows), diploid (indicated by white thick arrows), and tetraploid (marked by dashed line) cells with exclusively *P. ridibundus* chromosomes. Haploid spermatids with *P. ridibundus* chromosomes were labeled with black arrowheads. Somatic cells (pointed by arrowheads) included both *P. ridibundus* and *P. lessonae* chromosomes. Scale bar = 25 µm.

**Supplementary Figure S3. FISH with *RrS1* (green) and *PlesSat01-48* (red) probes on lampbrush chromosomal set from *P. esculentus* female from Slovakia*.*** *P. ridibundus* chromosomes are identified by the *RrS1* probe (green), whereas *P. lessonae* chromosomes are identified by the lack or decrease of signals from *RrS1* probe and the presence of signals from *PlesSat01-48* probe (red) in the pericentromeric regions of two small chromosomes. Chromosomes are counterstained with DAPI (blue). Lampbrush chromosomal set is represented by 13 bivalents corresponding to *P. ridibundus* chromosomes. Signals of only *RrS1* probe (indicated by arrows) were observed in centromeres of all chromosomes forming bivalents while no signal from *PlesSat01-48* probe was observed. Since the large size of the individual chromosomal spread, 10 images were merged. Scale bar = 50 µm.

**Supplementary Figure S4. Principal component analysis (PCA) of cytogenetic variables in *Pelophylax esculentus* males from three Central European populations.** PC1 (52.8% of variance) represents a gradient from *P. ridibundus* (positive scores) to *P. lessonae* genome transmission into gametes (negative scores). PC2 (19.4% of variance) reflects the degree of a mixture of both parental genomes in gametes in triploid individuals (positive scores) to aberrations in meiosis in diploid hybrids with chromosomal rearrangements (negative scores). Each point represents one individual; shape indicates karyotype: circles — diploid hybrids without chromosomal rearrangements; squares — diploid hybrids without chromosomal rearrangements; diamonds — triploid hybrids with chromosomal rearrangements. Colors indicate geographic origin: green — Poland; blue — Czech Republic; orange — Slovakia. Shaded ellipses denote 95% confidence regions per population. Diploid males without chromosomal rearrangements from all three populations cluster tightly at positive PC1 scores regardless of geographic origin, while diploid males with chromosomal rearrangements are displaced along the negative PC1 and PC2 axes. Triploid males from Slovakia are uniquely separated along the positive PC2 axis, reflecting their distinct meiotic phenotype.
